# Silicon application and related changes in soil bacterial community dynamics reduced ginseng black spot incidence in *Panax ginseng* in a short-term study

**DOI:** 10.1186/s12866-019-1627-z

**Published:** 2019-11-26

**Authors:** Meijia Li, Qiuxia Wang, Zhengbo Liu, Xiaoxi Pan, Yayu Zhang

**Affiliations:** Institute of Special Wild Economic Animals and Plants, Chinese Academy of Agriculture Sciences, Changchun, 130112 People’s Republic of China

**Keywords:** *Alternaria panax*, Silicon, Ginseng black spot, Soil bacterial community, *Panax ginseng*, Illumina HiSeq sequencing

## Abstract

**Background:**

This study analyzed the effect of silicon (Si) application on the occurrence of ginseng black spot caused by *Alternaria panax*. We explored the differences in soil physical and chemical factors and microbial community structure following Si application as well as the key factors that affected the occurrence of ginseng black spot in soil. Potted *Panax ginseng* plants were used to assess the effect of Si treatment on ginseng black spot. Soil physical and chemical properties were comprehensively analyzed. Bacterial communities were analyzed using Illumina HiSeq sequencing targeting the 16S rRNA gene.

**Results:**

After inoculation with *A. panax*, the morbidity (and morbidity index) of ginseng with and without Si was 52% (46) and 83% (77), respectively. Soil physical and chemical analysis showed that under the ginseng black spot inoculation, bacterial communities were mainly affected by pH and available potassium, followed by ammonium nitrogen and available Si. NMDS and PLS-DA analyses and the heat maps of relative abundance revealed that Si application elevated the resistance of ginseng black spot as regulated by the abundance and diversity of bacterial flora in rhizosphere soils. Heatmap analysis at the genus level revealed that *A. panax* + Si inoculations significantly increased the soil community abundance of *Sandaracinus*, *Polycyclovorans*, *Hirschia*, *Haliangium*, *Nitrospira*, *Saccharothrix*, *Aeromicrobium*, *Luteimonas*, and *Rubellimicrobium* and led to a bacterial community structure with relative abundances that were significantly similar to that of untreated soil.

**Conclusions:**

Short-term Si application also significantly regulated the structural impact on soil microorganisms caused by ginseng black spot. Our findings indicated that Si applications may possibly be used in the prevention and treatment of ginseng black spot.

## Background

Ginseng black spot, caused by *Alternaria panax* Whetz, is a common soil-borne disease and one of the most serious diseases affecting the above-ground parts, especially the leaves, of *Panax ginseng*. This pathogen is distributed widely in the Changbai Mountains of China and other ginseng production regions, and accounts for more than 20—30% of the annual incidence, which is very common in cultivated and wild ginseng. *Alternaria panax* infestation may lead to 10—20% yield loss of the total crop. Infection first appears as elongated reddish to dark brown crevices in the infected areas. In seedlings, the stems are gradually girdled and thus collapse, resulted in damping-off [1]. In older plants, foliar infections appear later in summer, characterized by rapidly enlarging dark brown necrotic spots (circular, ellipsoid, or wedge-shaped) surrounded by chlorotic margins.

Silicon (Si) has been demonstrated to play an important role in enhancing plant resistance to disease. Si deposition has been suggested to create a physical barrier along cell walls and prevent fungal penetration into the plant [2]. Additional studies have indicated that Si is related to plant-pathogen interactions for the control in diseases in different plant species [3], and aids in the enhancement of plant resistance against disease caused by viruses, fungi, bacteria, and nematodes. Recently, it was suggested that the deposition of Si in the apoplast may prevent fungal effectors from entering the target cells, thus altering the development of the pathogens [4]. Another recently study showed that Si treatment conferred an effective protection of soybean plants against *Phytophthora sojae* in a hydroponic experiment [5]. Agricultural soil productivity largely depends on microbial diversity and community composition, which significantly affects plant growth and crop quality [6]. The homeostasis of the soil microbial community can suppress pathogens and promote plant growth [7]. Plant—microbe interactions remodel the complex biological and ecological processes in soil, where roots are influenced by the rhizosphere [8]. Many studies have assessed the effect of Si on plant-microbe interactions and have demonstrated that Si enhances plant resistance to pathogens by activating defense reactions [9, 10]. Recently, a pot experiment demonstrated that Si addition decreased the concentrations of water-soluble and exchangeable arsenic in soil and, therefore, decreased the bioavailability of red soil arsenic in *Panax notoginseng* [11].

The present study, therefore, aimed to investigate if Si treatment would enhance the resistance of ginseng against *A. panax*. The study objectives were to evaluate the effect of Si on the prevention and treatment of ginseng black spot and to analyze the interaction between soil properties and plant growth responses. Further objectives were to determine the changes in the dynamics, i.e., the structure, composition, and abundance, of the soil microbial community in response to infection with *A. panax* and treatment with Si to determine the underlying factors that may influence the quantity and composition of soil bacteria.

## Results

### Disease index and incidence and plant weights

**Fig. 1:**
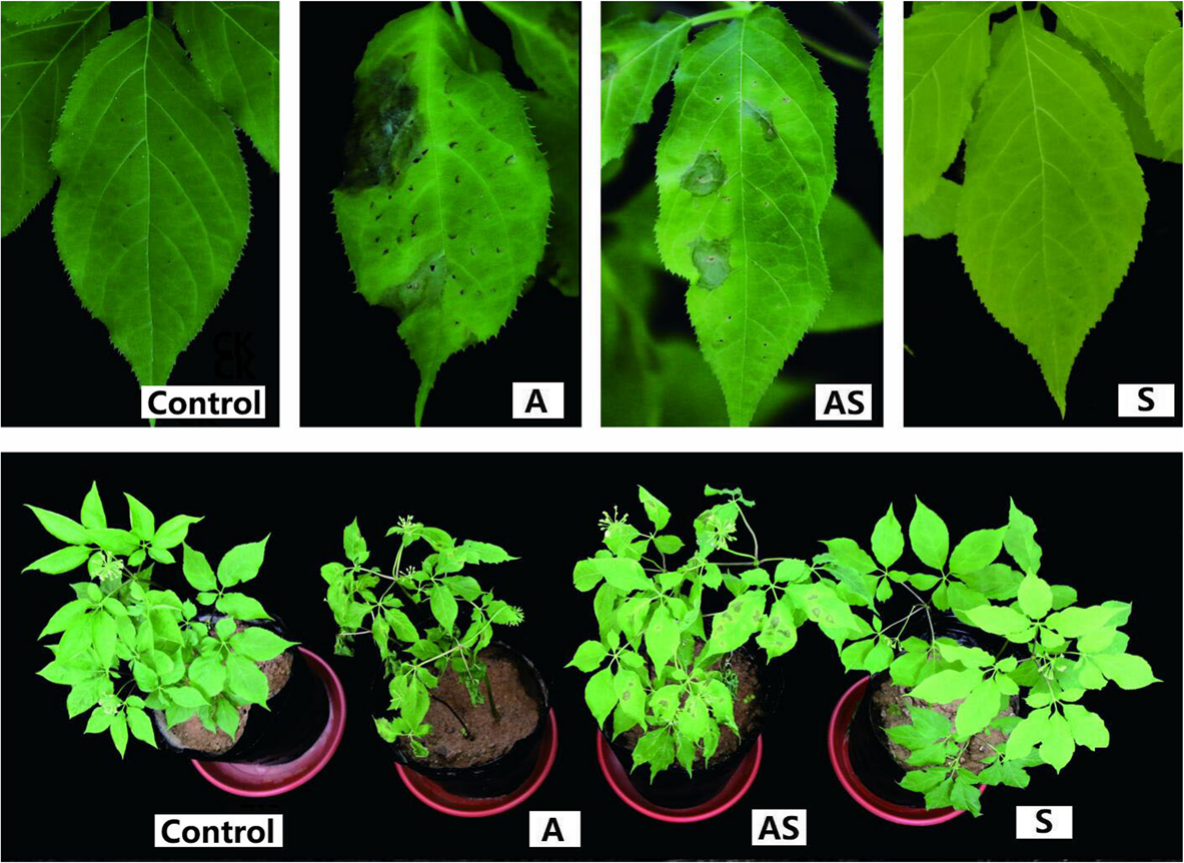
The effect in different treatments of soil. Abbreviations: CK, ginseng control plants; A, plants only inoculated with *A. panax*; AS, plants inoculated with *A. panax* + Si; S, plants only inoculated with Si

Figure 1 shows the phenotypic differences in leaves of *P. ginseng* in 9 dpi among treatment groups: Control, A, AS, and S. Significant differences were observed in the severity of *A. panax* infections under Si treatment (Fig. 1). As shown in Fig. 1, no effect of Si on biomass was observed compared to the Control group. The differences between Control plants and group S plants were not obvious, however, group AS plants were obviously healthier than group A plants (Fig. 1). The first symptom of leaf spots appeared soon (3 days) after post inoculation (dpi), followed by stunting and blight within a few days. As shown in Table 1, Si treatment significantly reduced the disease incidence and disease index of ginseng black spot.

**Table 1:** Effect of silicon application on the disease incidence and disease index of ginseng black spot

Treatment	Disease incidence (%)	Disease index
A	83.5 ± 6.5a	77.8 ± 7.5a
AS	52.6 ± 9.7b	46 ± 5.6b

Abbreviations: A, *A.panax* inoculated ginseng; AS, Silicon inoculated in soil with *A. panax* infection

There was no significant difference in dry weight among non-inoculated (pathogen) plants: Control plants (1.12 ± 0.81 g) and group S plants (1.23 ± 0.59 g). However, the plant dry weight was significantly reduced in group A plants. Apparently, Si treatment resulted in significantly heavier plants (Table 2). After 9 days post-treatment, the fresh weight of group AS plants was 15% higher than that of group A plants (Table 3).

**Table 2:** The fresh weight and dry weight of the ginseng after different treatments

Treatment	fresh weight	dry weitht
CK	5.53 ± 1.25a	1.12 ± 0.81a
A	4.329 ± 1.35b	0.56 ± 0.12b
AS	5.12 ± 1.36a	0.97 ± 0.24b
S	5.67 ± 1.28a	1.23 ± 0.59a

**Table 3:** The fresh weight and dry weight of ginseng shoots and ginseng roots after different treatments

	ginseng shoots		ginseng roots	
Treatment	fresh weight	dry weitht	fresh weight	root dry weight
CK	2.51 ± 0.21a	0.38 ± 0.11a	3.02 ± 1.21b	0.75 ± 0.12b
A	1.56 ± 0.12d	0.20 ± 0.02d	2.57 ± 0.89d	0.52 ± 0.11c
AS	2.23 ± 0.25c	0.26 ± 0.13c	2.89 ± 0.98c	0.61 ± 0.12b
S	2.39 ± 0.31b	0.35 ± 0.13b	3.28 ± 1.02a	0.79 ± 0.19a

Different letters within a column indicates significant difference among treatments *p* < 0.05

### Soil properties and plant growth responses

Soil properties are presented in Table 4. A one-way ANOVA showed that the treatments significantly recovered the soil property parameters from disease treatment (*P* < 0.05). (Table 4). The pH value of the Control soil samples was ~ 7.39. Compared with the Control group, the soil pH, NO_3_^−^-N, and NH_4_^+^-N were significantly reduced in Group A (*P* < 0.05). In contrast, the ratio of available P and available K (*P* < 0.05) were significantly increased. Furthermore, AS significantly increased the soil pH, and NO_3_^−^-N and NH_4_^+^-N contents (*P* < 0.05), and significantly reduced the ratio of available P and available K (*P* < 0.05) compared to the A treatment, i.e., without Si (*P* < 0.05). No significant differences in the above-mentioned nutrients, except available Si, was detected between group S and Control.

**Table 4:** Characteristics of soils after different treatments

Indexes	CK	A	AS	S
pH	7.39 ± 0.12ab	7.05 ± 0.24c	7.39 ± 0.26a	7.38 ± 0.36b
Available phosphorus AP (mg/kg)	13.15 ± 2.61ab	14.95 ± 3.25a	12.25 ± 2.68b	12.73 ± 2.62b
Available potassium AK (mg/kg)	194.48 ± 3.26b	217.15 ± 2.35a	187.64 ± 3.69b	199.06 ± 2.65b
Ammonium nitrogen NH_4(g/kg)_	16.42 ± 2.35a	4.58 ± 1.02c	9.38 ± 2.36b	14.38 ± 1.25a
Nitrate nitrogen NO_3(g/kg)_	1.31 ± 0.25	1.15 ± 0.36	1.89 ± 0.28	1.97 ± 0.69
Available silicon ASi (mg/kg)	457.99 ± 20.35b	451.78 ± 25.69b	457.02 ± 19.89b	492.06 ± 26.98a

Different letters within a column indicates significant difference among treatments *p* < 0.05

### Analysis of bacterial composition and diversity of soil bacterial community structure based on 16S rRNA gene sequencing

Bacteria-targeted regions were completely amplified by PCR and fully sequenced for all soil samples. The raw sequence libraries were screened to remove reads that originated from sequencing noise or putative chimeric sequences. Using the 12 soil samples from the different treatments, a total of 815,609 valid 16S rDNA sequences were obtained by filtering and processing according to a 97% similarity. Variation of a single soil sample ranged from 56,510 to 76,384 sequences, and the above sequences were retained for further analysis.

The effective sequence number and OTU number of each group of samples did not significantly differ between the treatment groups and the Control group, as shown in Table 5. The sequencing coverage of samples ranged from 98.5 to 98.6%. After sample diversity (alpha diversity) analysis, the indexes reflecting the abundance and diversity of microbial communities were calculated, and the results of all treatments were analyzed using a one-way ANOVA (Table 5). The coverage index of the sample library was more than 98.5%, which indicated that the sequencing results represented the real situation of the bacterial population in the sample. The microflora richness index (Chao1, ACE) and biodiversity index (Shannon, Simpson) of the samples revealed that the diversity of the bacterial populations in the soil samples was relatively high (Table 5). Further analysis revealed that at a 97% similarity level, the Shannon index and Simpson index of soil bacteria of each treatment group were not significantly different from those in the Control group.

**Table 5:** The bacterial diversity indices of ginseng rhizosphere soil samples with different treatment

	Sequence number	OTU	Coverage %	Shannon	Simpson	ACE	Chao1
CK	207,437	7381	98.6	11.2 ± 0.2	0.9989 ± 0.01	5250.6 ± 23.6a	5149.3 ± 123.3a
A	209,093	7210	98.6	11.1 ± 0.3	0.9989 ± 0.02	5048.9 ± 69.7b	5007.6 ± 369.9b
AS	206,227	6974	98.5	11.1 ± 0.2	0.9988 ± 0.01	5138.2 ± 102.3a	5063.6 ± 325.3b
S	192,852	7063	98.5	11.1 ± 0.1	0.9988 ± 0.02	5141.5 ± 69.8a	5163.2 ± 345.6a

Different letters within a column indicates significant difference among treatments *p* < 0.05

### Analysis of soil bacterial community structure

**Fig. 2:**
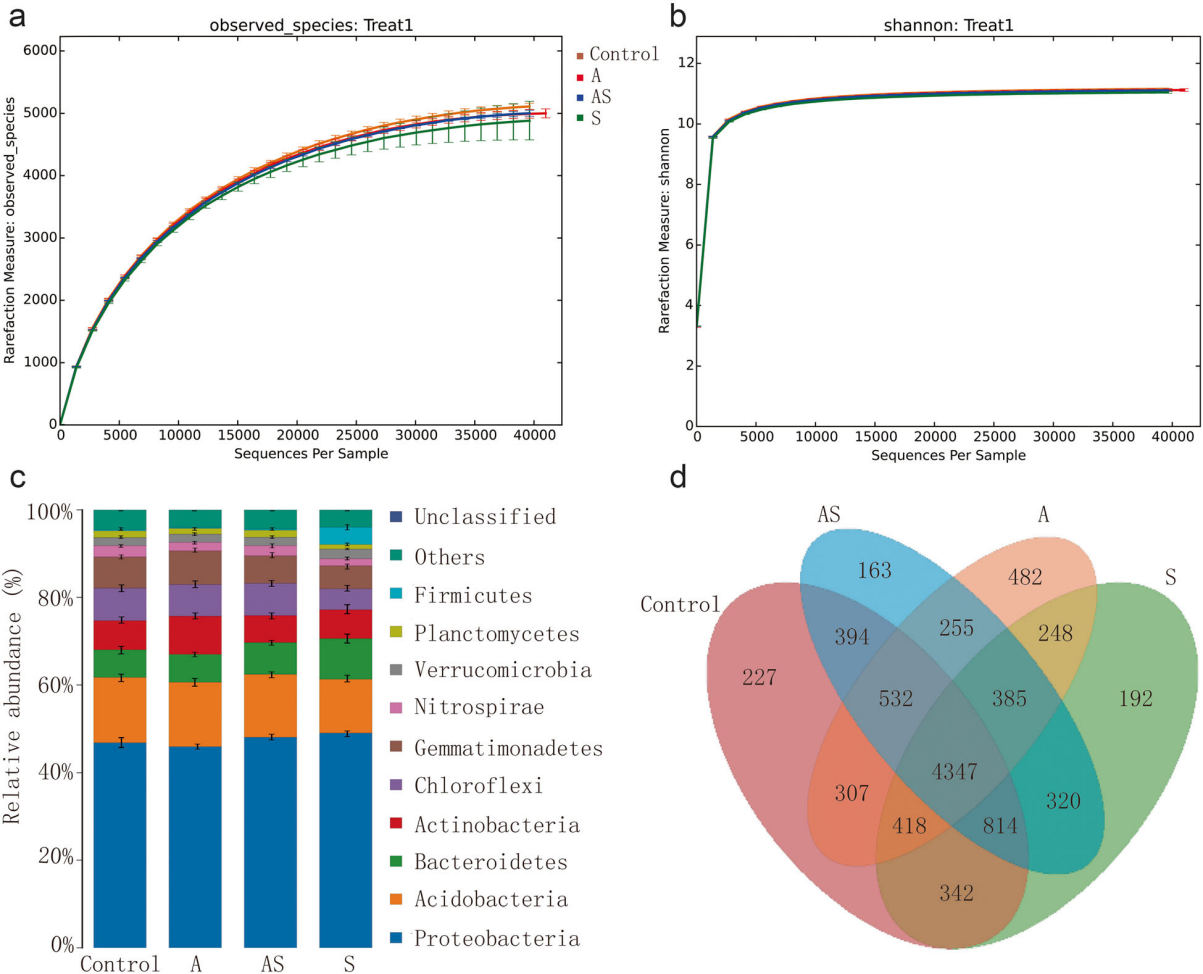
Overall analysis of bacterial communities in different treatments of soil. **a** The bacterial composition of in different treatments of soil at the phylum taxonomic level. **b** The Venn map of bacterial communities in different treatments of soil. **c** The bacterial composition of in different treatments of soil at the phylum taxonomic level. **d** The Venn map of bacterial communities in different treatments of soil

**Fig. 3:**
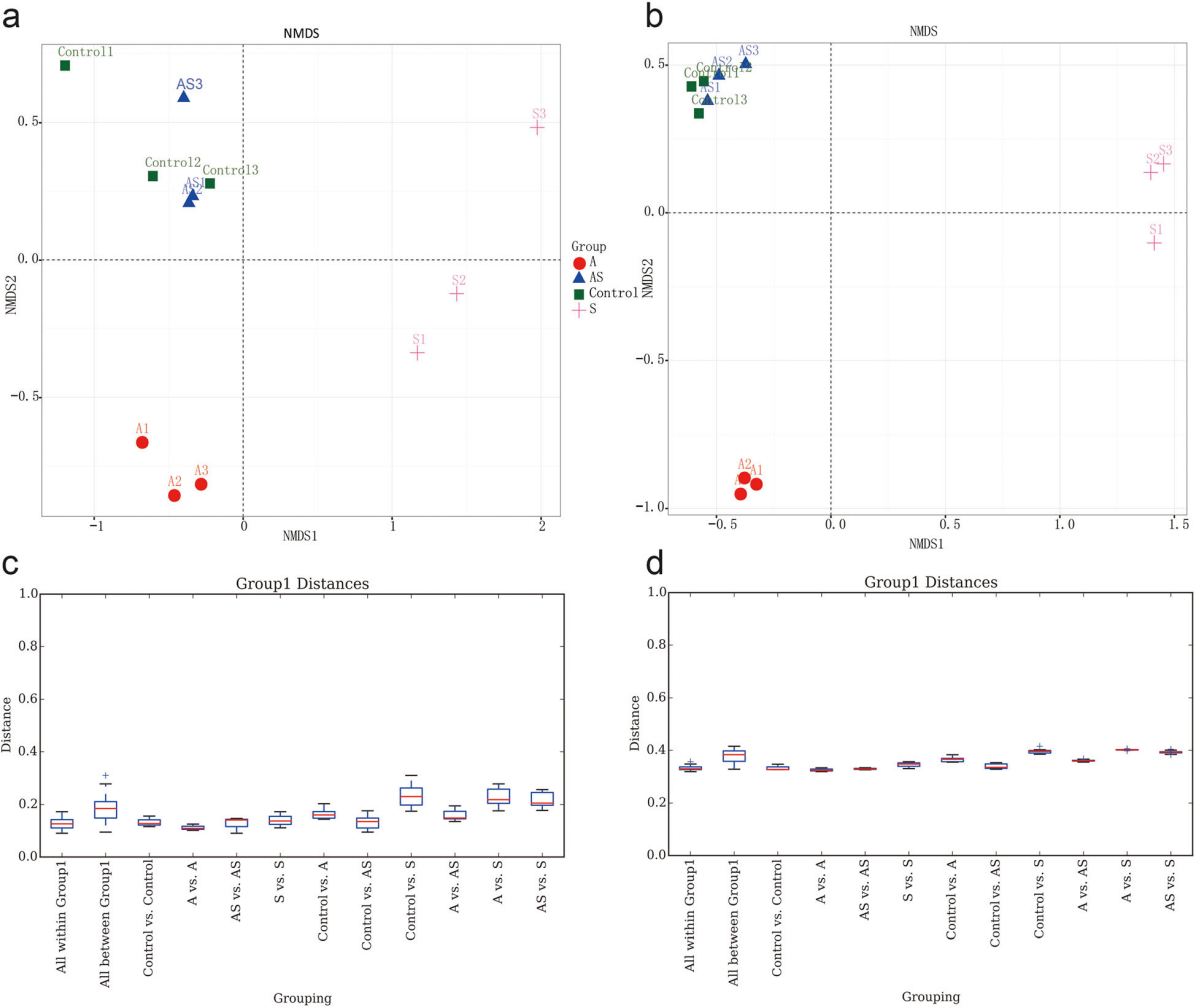
NMDS analysis of bacterial community diversity in different samples (**a**) Non-metric multidimensional scaling (NMDS) plots of operational taxonomic unit tables from all substrates based on abundances of bacterial community similarities using unweighted unifrac-distance of matrix (**b**) Unweighted weighted unifrac-distance box-line graph. **c** NMDS plots of operational taxonomic unit tables from all substrates based on abundances of bacterial community similarities using weighted unifrac-distance of matrix (**d**) Weighted unifrac-distance box-line graph

According to the abundance of bacterial OTU types in the 12 soil samples, a non-metric multidimensional scale (NMDS) diversity analysis was conducted to determine the differences in the bacterial compositions of the different samples and treatments (Fig. 2). The NMDS results were evaluated using the UniFrac distances to estimate the phylogenetic relatedness among the bacterial communities (Fig. 3a, c). The soil bacterial communities were found to be totally distinct between groups A and S, i.e., when treated with *A. panax* or Si (NMDS). Among treatment groups, the soil bacterial composition of group Control was most similar to that of group AS, i.e., had the highest phylogenetic relatedness, and the group AS bacterial flora could be independently distinguished from that in the infected soil (group A). However, the composition of bacterial flora in group S differed from that of the other treatments. In summary, Si application significantly regulated the changes in bacterial flora (back to the composition of Control) that were induced by inoculation of ginseng black spot (group AS).

### Cluster analysis of soil bacterial community structure

**Fig. 4:**
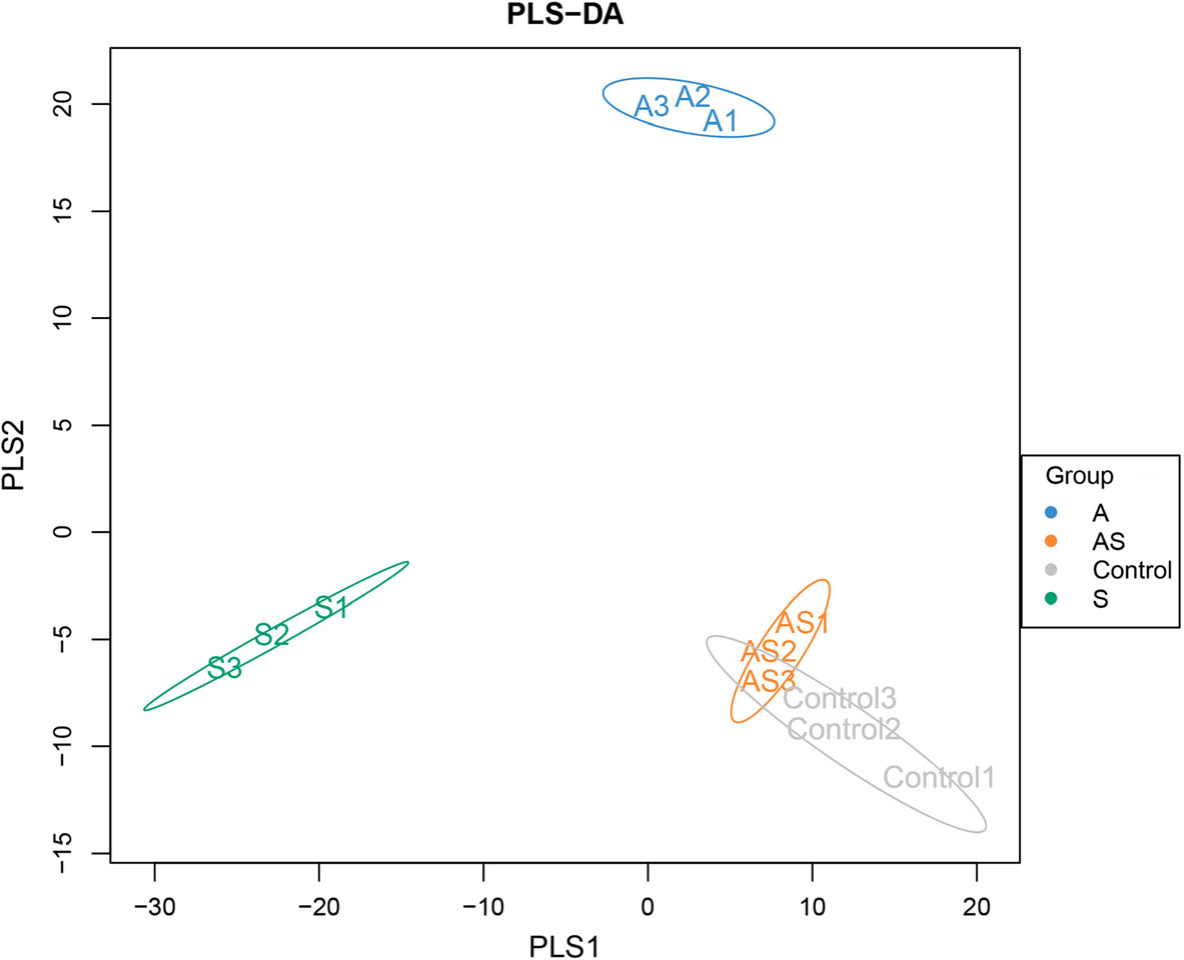
PLS-DA analysis of different soil samples

Based on a Beta diversity analysis, a distance matrix was obtained for the 12 soil samples, and a hierarchical clustering analysis was conducted using the unweighted group average method (UPGMA) (Fig. 3b, d). The soil samples of groups S and AS were classified as one branch, and those of groups Control and AS group were classified as one branch. The results were consistent with those of the NMDS analysis, which fully demonstrated that the soils inoculated with Si + ginseng black spot (AS group) were significantly recovered compared with the soils inoculated with only ginseng black spot (S group). PLS-DA analysis showed that the microbial composition of soil in the AS group was significantly altered following Si treatment. The results suggested similarities between group Control and AS, but not with nor among the other two groups. In summary, Si was again shown to have alleviated the changes in soil bacteria caused by ginseng black spot (Fig. 4).

### Heat map analysis of the soil bacterial community structure

**Fig. 5:**
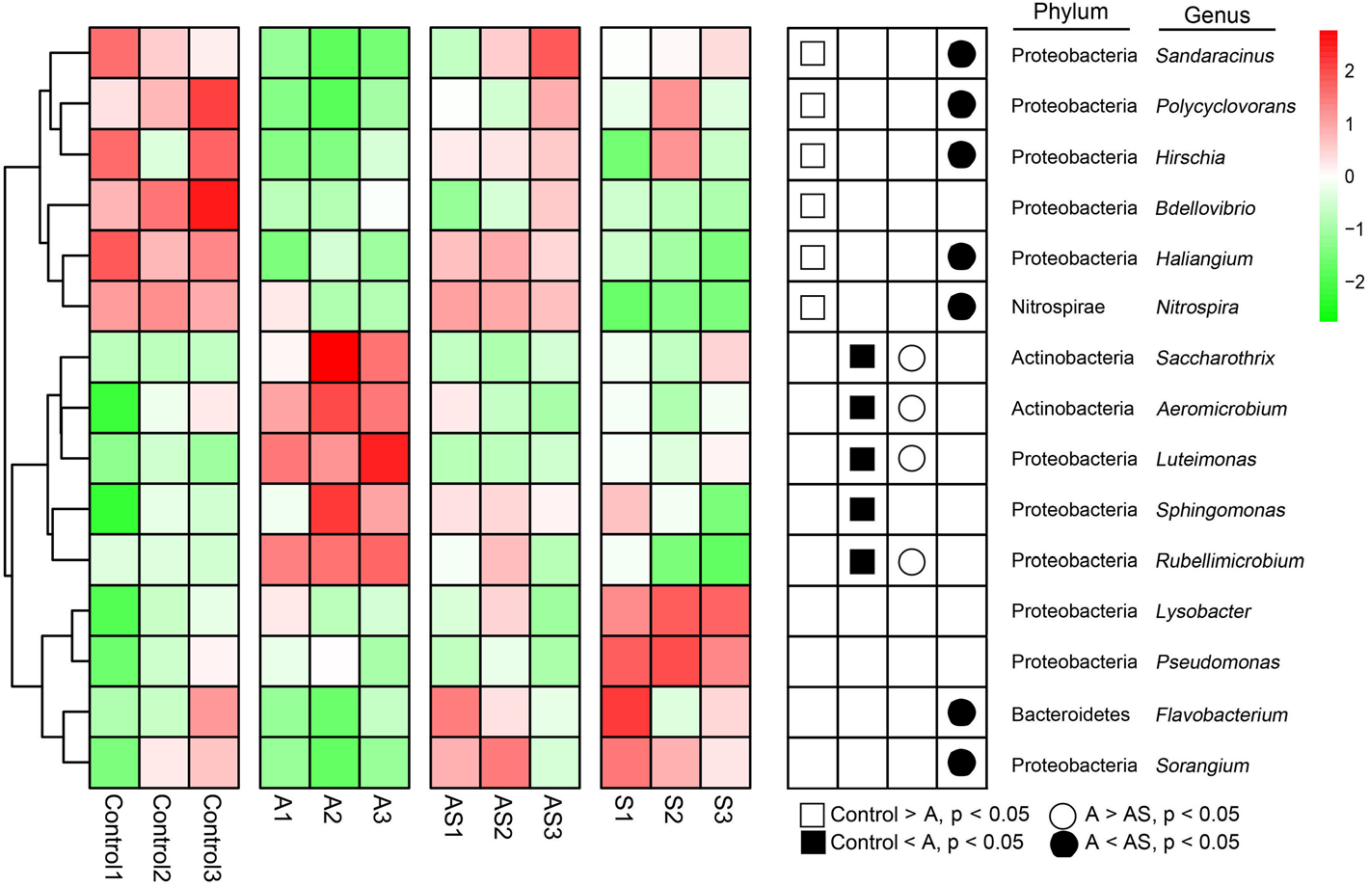
Heat map comparison of the dominant bacterial with average relative abundance from blue to red means relative abundance from low to high

A heat map of the bacterial community structure among different samples (Fig. 5) revealed the relative abundances of the various bacterial groups (at phylum and genus levels) and that significant differences were observed among different groups of samples. The results showed that, at the phylum level, Proteobacteria, Nitrospirae, Actinobacteria, and Bacteroidetes were the four main groups (Fig. 5). The relative abundances (represented by the color depth in Fig. 5) of *Sandaracinus*, *Polycyclovorans*, *Hirschia*, *Bdellovibrio*, *Haliangium*, and *Nitrospira* were significantly higher in group Control than those of group A (*P* < 0.05). In addition, the relative abundances of *Sandaracinus*, *Polycyclovorans*, *Hirschia*, *Haliangium*, and *Nitrospira* in group AS were significantly higher than those in A (P < 0.05). The results showed that Si application significantly regulated the structural impact of soil microorganisms caused by ginseng black spot inoculation.

### Factors influencing the quantity and composition of soil bacteria

Correlation analysis showed (Table 6) that most of the other dominant bacterial groups had significant correlations with soil chemical properties, except *Arenimonas*, *H16*, and *RB41*, which showed no correlations with all chemical indicators. *Haliangium* and available K were significantly negatively correlated; *Phenylobacterium* (phenyl coli) was very significantly negatively correlated with pH and *Gemmatimonas* (bacillus); *Nitrospira* (nitrification spirillum) was negatively correlated with NO_3_^−^-N; *Mesorhizobium* (rhizobia) was very significantly positively correlated with NO_3_^−^-N; *Gemmatimonas* (bacillus), *Nitrospira* (nitrobacteria), and available Si were significantly negatively correlated; *Lactobacillus* (lactobacillus), *Mesorhizobium* (rhizobium), and available Si were significantly positively correlated. *Haliangium* was significantly positively correlated with pH.

**Table 6:** Pearson’s correlation coefficients between various physicochemical variables and the relative abundances of main genera (> 1%) across all samples

	pH	AP	AK	NH_4_^+^-N	NO_3_^−^-N	ASi
Arenimonas	0.21	−0.33	− 0.21	− 0.03	0.57	0.50
Gemmatimonas	− 0.40	0.27	0.41	0.05	−0.66*	−0.62*
H16 (Proteobacteria)	0.3	0.01	−0.19	0.50	−0.38	−0.23
Haliangium	0.65*	−0.21	−0.67*	0.50	−0.45	− 0.27
Lactobacillus	0.13	−0.37	−0.01	0.09	0.96**	0.92**
Mesorhizobium	−0.01	−0.36	0.01	0.05	0.80**	0.84**
Nitrospira	0.48	−0.02	0.51	0.26	−0.69*	−0.61*
Phenylobacterium	−0.72**	0.42	0.72**	−0.07	0.04	0.03
RB41 (Acidobacteria)	0.04	0.10	0.11	0.06	NO_3_^−^-N	−0.29

Values in bold indicate significant correlations at **p < 0.01 and *p < 0.05

## Discussion

### Silicon reduced disease incidence and disease index of ginseng black spot

Si has been shown to effectively improve the mechanical and physiological capacities of plants and enhance plant resistance to overcome various biotic and abiotic stresses [12, 13]. To examine the effect of Si application on ginseng black spot-infected plants, a pot experiment was performed with pretreatment of Si for 2 weeks, following 9 dpi with *A. panax.* Si application significantly reduced the disease incidence and index of ginseng black spot (Table 1) and clearly alleviated the incidence of leaf blight caused by *A. panax* (Fig. 1). Similarly, Si has been reported to enhance plant resistance to diseases, potentially through interacts with several key factors of the stress signaling pathway [14]. In comparison to group A plants, Si application was shown to increase the accumulation of shoot and root biomass in group AS *P. ginseng* plants. These findings suggest that Si triggered plant-microbial response mechanisms that directly limited ginseng black spot index and incidence in the leaves and thus enhanced *P. ginseng* biomass accumulation. However, the root and shoot biomass of group S plants was not significantly different compared with Control, which opposes the notion that Si promotes plant biomass accumulation [15–17]. In the present study, a short-term pot experiment was used to determine the effects of Si application on ginseng black spot and the soil bacterial community, however, future research is needed to clarify the effects of long-term applications on Si-*P. ginseng*-soil interactions.

### Soil properties

Compared with the Control, inoculation of ginseng black spot (in group A) significantly reduced the soil pH and NH_4_^+^-N content, and significantly increased the ratio of available K. After Si application, the reduced soil pH and NH_4_^+^-N content were significantly recovered to the levels of group Control. Compared with group A, the ratio of available P and available K was reduced in group AS, which resulted in similar soil physical and chemical indexes to that of group CK. In the present study, Si application led to the amendment of the soil pH changes caused by the inoculation of ginseng black spot. However, the soil pH of group Control and group S were not significantly different, and thus Si application alone did not alter the pH. A similar result was found in a study with *P. notoginseng*, whereby Si increased the soil pH when in the presence of arsenic, which may have been because the Si treatment decreased the bioavailability of arsenic [11]. Although our study does rule out the possibility of other chemical differences among treatments, our data doe does suggest that nutrient availabilities were not the driving differences in soil properties without pathogen inoculation. It is important to consider plant root exudates and their great impact on the population and community structure of soil microbes [18]. In our study, Si application may have affected the root exudates, and other root-derived molecules; as was observed in another study when plants were infected by a fungus [19]. Further research is needed to elucidate the root exudates-Si, plants-Si, and root exudates-plants interactions in the soil-*P. ginseng* system. However, besides available Si, there were no significant differences in the above-mentioned nutrients between groups S and Control. Therefore, it is likely that Si altered the root exudes rather than physicochemical soil characteristics, which caused the recovery of the bacterial community from A to AS, which was similar to Control.

### Soil microbial community composition and diversity

Microbial community diversity is an important component of soil health [20]. The impact of Si on bacterial richness and diversity was analyzed by high-throughput sequencing. The soil bacterial richness (Shannon index and Simpson index) was not significantly different under the different treatments (Fig. 2), which indicated that Si and *A. panax* treatments did not alter the number of bacterial species in the short-term. However, genes-level differences were found in the relative abundances of bacterial species. Interestingly, the relative abundances of *Saccharothrix*, *Aeromicrobium*, *Luteimonas*, and *Rubellimicrobium* recovered (*P* < 0.05) from lower levels in group A to higher levels (similar to Control) following Si application (Fig. 5). The results showed that Si application significantly regulated the structural impact of soil microorganisms caused by ginseng black spot. *Aeromicrobium* as a potential disease suppression indicator [21, 22] and a member of phylum Actinobacteria. Moreover, the antibiotics produced by Actinobacteria are able to suppress various plant diseases [23, 24]. Disease-suppressive natural soils, with reference to a variety of agricultural crop diseases, have been reported for wheat Take-all and Rhizoctonia bare patch diseases [25], Fusarium wilt on strawberry and vanilla [26], and *Rhizoctonia solani* on sugar beet [27]. This characteristic relates to the abundance of certain beneficial soil microbes [26, 27], which produce antimicrobial compounds that directly inhibit pathogens. In addition, indirect pathogen inhibition via induced systemic resistance (ISR) may occur, via the triggering of plant immune responses [28]. However, in the event of a severe disease outbreak, consecutive cropping cycles of the same species are stipulated for disease-suppressive microbes to flourish. The proposed hypothesis suggests that, when invaded, certain favorable microbes are amplified and sustained in plants [25, 29, 30]. The bacterial composition of the group Control soil was similar to that of group AS (Si-treated), i.e., their compositions had the highest phylogenetic relatedness. The group AS bacterial flora differed from that of the group A, and the composition of bacterial flora in group S differed from that of the other treatments. The results showed that Si application significantly regulated the changes in bacterial flora caused by ginseng black spot inoculation, and increased the levels in group AS to almost the same as group Control. Recent reports additionally revealed that *Arabidopsis* plants can stimulate specific favorable microbes in the rhizosphere, even in natural soils [31]. Another validating instance was seen when a *Xanthomonas* sp., *Stenotrophomonas* sp., and *Microbacterium* sp. beneficial consortium was activated in the rhizosphere as part of the downy mildew *Hyaloperonospora arabidopsidis* induced foliar defense [19]. Furthermore, these strains, when isolated, collectively induced downy mildew resistance, when inoculated back into *Arabidopsis*; and interestingly, the resistance of a second plant population grown in the same soil was considerably amplified as a result of the downy mildew infection in the first population. These outcomes collectively suggested that beneficial microbes ensue from plant invasions, which in turn prompt a memory or “soil-borne legacy” that amplifies the next plant generation defenses against harmful pathogens [31–34]. The implication here is that Si triggered the soil bacterial community response, which might have directly regulated plant growth. In the present study, we observed that the bacterial community differed in group AS compared with group A, i.e., between Si and no Si treatments. Overall, the increase in the soil bacterial diversity after Si application may contribute to the suppression of ginseng black spot disease. The functionality of root exudates and other root-derived molecules, is indicated in this process [31, 32, 35–37]; albeit this hypothesis requires validation. The most recent research, however, also reports that plants secure favorable rhizosphere communities via the modification of plant exudation patterns, induced by exposure to aboveground pathogens, which subsequently benefits future plant generations [19].

In summary, Si can alter the structure and diversity of the soil microbial community by directly and indirectly affecting the growth of plants, and the altered soil microbial community can, in turn, affect the plants [38–40].

## Conclusion

This study provided a detailed outline of the bacterial community compositions in Si applications inoculated with ginseng black spot using Illumina HiSeq sequencing. Si application of ginseng black spot-inoculated plants significantly optimized soil bacterial population structure, improved soil bacterial activity and diversity, and thus effectively prevented and controlled the occurrence of ginseng black spot. In addition, we speculated that Si indirectly altered the structure, composition, and abundance of the soil microbial community by directly altering the root exudates or inducing plant systemic resistance. In conclusion, the present study demonstrated the good application prospect of Si and that it is recommended for use as ginseng fertilizer for the prevention and treatment of ginseng black spot.

## Methods

### Experimental design

Two-year-old fresh ginseng roots (*Panax ginseng* Meyer) were provided by dongdu ginseng technology development co., LTD in April 2017 and placed in sand at 23 °C. After 6 days, the roots sprouted and were then washed with deionized water, and transplanted into PVC pots (120 × 180 mm, diameter × height) containing turfy soil (6 seedlings per pot). The ginseng seedlings were grown under greenhouse conditions: temperatures of 17–28 °C, a relative humidity of 70%~ 80%, and a 14 h photoperiod. Before *A. panax* inoculation, half of the plants were pretreated for 2 weeks with potassium silicate (pH = 7.0) as the Si source. After Si pretreatment, the plants were inoculated with conidia of the appropriate *A. panax* pathogen. The conidia of *A. panax* infecting *P. ginseng* were identified by PCR of the internal transcribed spacer (ITS) region generating 553~554 bp fragments and the glyceraldehyde 3-phosphate dehydrogenase (gpd) for 565~566 bp fragments, respectively. Sequence showed 100% identical to that of *A. panax* (JF417572 of ITS, JF417653 of gpd). The *A. panax* strain was deposited in the Culture Collection Center of Yangtze University in Jingzhou, China*.* Spores were flushed from colonies and then resuspended in sterile distilled water at 1 × 10^5^ spores/mL. The sterilized surfaces of detached spring ginseng leaves were inoculated with 20 μL conidial suspension and incubated under the same greenhouse conditions for 9 days, when black spot symptoms became visible on the leaves.

Plants were grown under four kinds of treatment: ginseng control plants (Control), plants only inoculated with *A. panax* (A), plants inoculated with *A. panax* + Si (AS), and plants only inoculated with Si (S), with 18 plants (3 pots) per treatment. To test the prophylactic role of Si, the Si concentration was set at 1.7 mM, i.e., the highest possible concentration of silica acid in solution [4].

Six seedlings of ginseng were randomly selected from each treatment group, and the soils were mixed to form a single representative sample. After inoculation with *A. panax* for 9 days, plants were removed from the soil and the excess soil was carefully shaken off. The rhizosphere soil (i.e., adhering to the roots) was collected as previously described by Bulgarelli et al. [41], with some modifications. Three replicate rhizosphere soil samples were obtained per treatment. Soil samples (*n* = 12) were air-dried for 2 weeks, passed through a 2 mm sieve, and stored at − 80 °C.

### Plant dry weights and analysis of disease index and incidence

For *A. panax* infected plants, ginseng black spot incidence was recorded from 9 days after *A. panax* inoculation. The 18 plants (3 pots) per treatment were collected to calculate the percentage of diseased plants and count disease index, using the following equations [42]:

$$ Disease\ incidence= the\ number\ of\ diseased\ plants/ the\ total\ number\ of\ plants\times 100\% $$

$$ Disease\kern0.17em index=\sum \left(A\times B\right)\times 100/\sum \times 4 $$

where A is the disease class (0, 1, 2, 3, 4) and B is the number of plants in the corresponding disease class.

For each plant, the shoots and roots were separated and weighed after air drying (dry weight, g) for 2 weeks at 30 °C.

### Sampling and chemical analysis

Air-dried plants and soil samples were used in the nutrient analysis. About 50 mg oven-dried plant tissue was digested with a mixture of 8 mL HNO_3_ and 2 mL HClO_4_ at 200 °C for 120 min in a semi-closed system. The digestates were cooled down to 25 °C and made up to 50 mL with 4% (v/v) HNO_3_ solution. The soil pH (1:5, soil: water) was measured using a glass electrode (SK220, Switzerland). Soil nitrate nitrogen (NO_3_^−^-N) was assayed using a continuous flow analytical system (SJAKAR SAN^++^, The Netherlands). Ammonium nitrogen (NH_4_^+^-N) in the soil was extracted with 0.01 M CaCl_2_, and the concentration was measured by an Auto Analyzer (Auto Analyzer 3, Germany). Potassium (K) in the soil was dissolved with ammonium acetate and calculated by flame photometry. Soluble phosphorus (P) was dissolved with sodium bicarbonate and its concentration measured using the molybdenum blue method [43].

### High-throughput sequencing

The total DNA was extracted from 0.5 g of each soil sample using a bacterial DNA Isolation Kit (Omega Bio-tek, Norcross, GA, USA) following the manufacturer’s instructions [44]. To assess the bacterial community composition, Illumina HiSeq platform (Illumina, San Diego, California, USA) was used in present study. The quantity and quality of extracted DNAs were measured using a Nanodrop 1000 (Thermo Fisher Scientific, Wilmington, DE, USA) and agarose gel electrophoresis, respectively. Primers for amplification and preamplification sequence: bacterial 16S rRNA gene V3-V4 region primers: 338F (5′-ACTCCTACGGGAGGCAGCA-3′) and 806R (5′-GGACTACHVGGGTWTCTAAT-3′). DNA was amplified by PCR under conditions of 95 °C 2 min, followed by 27 cycles of 95 °C extends 30 s, 55 °C for 30s and 72 °C for 45 s; and a final extension at 72 °C for10 min, then maintained at 10 °C until halted. The PCR reactions were performed triplicate in a 20 μL mixture contained 4 μL 5 Mix × FastPfu Buffffer, 2 μL of 2.5 mM dNTPs, 0.4 μL of each primer (5 μM), 0.4 μL of TransStart FastPfu DNA Polymerase (TransGen Biotech, Beijing, China), and 10 ng of template DNA [45]. PCR amplicons were purified with A gencourt AMPure Beads (Beckman Coulter, Indianapolis, IN) and quantified using the PicoGreen dsDNA Assay Kit (Invitrogen, Carlsbad, CA, USA). After the individual quantification step, amplicons were pooled in equal amounts, and pair-end 2 × 300 bp sequencing was performed using the Illumina HiSeq platform (Illumina, San Diego, California, USA) at Biomarker Technologies, Beijing, China.

The Quantitative Insights Into Microbial Ecology (QIIME, v1.8.0) pipeline was employed to process the sequencing data [46]. The low-quality sequences were filtered through the criteria [47, 48]. Paired-end reads were assembled using FLASH [49]. After chimera detection, the remaining high-quality sequences were clustered into operational taxonomic units (OTUs) at 97% sequence identity by UCLUST [50]. A representative sequence was selected from each OTU using default parameters. OTU taxonomic classification was conducted by BLAST searching the representative sequences set against the Greengenes Database [51]. Each OUT in each sample and the taxonomy was recorded in an OTU table, and OTUs containing less than 0.001% of total sequences across all samples were discarded. Sequences were deposited at the NCBI Short Read Archive and accession numbers are SRR9822023-SRR9822034.

Sequence data analyses were mainly performed using QIIME and R packages (v3.2.0). OTU-level alpha diversity indices, were calculated in QIIME. Beta diversity analysis was performed to investigate the structural variation of microbial communities across samples using UniFrac distance metrics [52, 53] and nonmetric multidimensional scaling (NMDS) [54]. Venn diagram was generated to visualize the shared and unique OTUs among groups using R package [55]. Taxa abundances at the phylum, class, order, family, genus and species levels were statistically compared among groups by Metastats [56]. PLS-DA (Partial least squares discriminant analysis) was also introduced as a supervised model to reveal the microbiota variation among groups, using the “plsda” function in R package “mixOmics” [48].

### Data analysis

One-way analysis of variance (ANOVA) was used to calculate the difference between treatments with variable soil pathogen abundance. The significance threshold was set at 0.05. The statistical analyses was performed using SAS 9.1 software (SAS Institute Inc., Cary, NC).

## Data Availability

The dataset(s) supporting the conclusions of this article are available in the following repositories: The raw read sequences were deposited at the NCBI Short Read Archive and accession numbers are SRR9822023-SRR9822034.

## Abbreviations

ANOVA: Analysis of variance
dpi: Days after post inoculation
gpd: Glyceraldehyde 3-phosphate dehydrogenase
ITS: Internal transcribed spacer
NMDS: Non-metric multidimensional scale
OTU: Operational taxonomic unit
PLS-DA: Partial least squares discriminant analysis
Si: Silicon
UPGMA: Unweighted group average method
